# Automatic detection of anomalies in screening mammograms

**DOI:** 10.1186/1471-2342-13-43

**Published:** 2013-12-13

**Authors:** Edward J Kendall, Michael G Barnett, Krista Chytyk-Praznik

**Affiliations:** 1 Discipline of Radiology, Janeway Child Health Centre, Memorial University of Newfoundland, Newfoundland A1B 3V6, Canada; 2 Prairie North Health Region, Battlefords Office, 1092 107th Street, North Battleford, Saskatchewan S9A 1Z1, Canada; 3 Radiation Oncology Department, Nova Scotia Cancer Centre, 5820 University Avenue, Halifax, Nova Scotia B3H 1V7, Canada

## Abstract

**Background:**

Diagnostic performance in breast screening programs may be influenced by the prior probability of disease. Since breast cancer incidence is roughly half a percent in the general population there is a large probability that the screening exam will be normal. That factor may contribute to false negatives. Screening programs typically exhibit about 83% sensitivity and 91% specificity. This investigation was undertaken to determine if a system could be developed to pre-sort screening-images into normal and suspicious bins based on their likelihood to contain disease. Wavelets were investigated as a method to parse the image data, potentially removing confounding information. The development of a classification system based on features extracted from wavelet transformed mammograms is reported.

**Methods:**

In the multi-step procedure images were processed using 2D discrete wavelet transforms to create a set of maps at different size scales. Next, statistical features were computed from each map, and a subset of these features was the input for a concerted-effort set of naïve Bayesian classifiers. The classifier network was constructed to calculate the probability that the parent mammography image contained an abnormality. The abnormalities were not identified, nor were they regionalized.

The algorithm was tested on two publicly available databases: the Digital Database for Screening Mammography (DDSM) and the Mammographic Images Analysis Society’s database (MIAS). These databases contain radiologist-verified images and feature common abnormalities including: spiculations, masses, geometric deformations and fibroid tissues.

**Results:**

The classifier-network designs tested achieved sensitivities and specificities sufficient to be potentially useful in a clinical setting. This first series of tests identified networks with 100% sensitivity and up to 79% specificity for abnormalities. This performance significantly exceeds the mean sensitivity reported in literature for the unaided human expert.

**Conclusions:**

Classifiers based on wavelet-derived features proved to be highly sensitive to a range of pathologies, as a result Type II errors were nearly eliminated. Pre-sorting the images changed the prior probability in the sorted database from 37% to 74%.

## Background

Breast cancer is the most common form of cancer among Canadian women, and is second only to lung cancer in mortality [1-3]. Women in higher risk groups, are encouraged receive a screening x-ray mammogram every two years, with further screening for very high risk patients, such as those with familial history or genetic predisposition.

Treatment efficacy is linked to early detection of tumors. The challenge in x-ray mammography is that features associated with pathology may be patent or subtly represented in the image. For example, micro-calcifications sometimes signal the presence of cancer. Due to calcium’s relatively high absorption of x-ray photons they appear as small bright regions in the mammogram and readily detected by CAD and human reviewers [4-8]. On the other hand, masses are evident in an x-ray if their density differs from that of the surrounding tissue, and this is often not the case. Masses may have almost any size, shape or structure [4,7,9-17]. Occasionally, masses are evident only by inducing deformation of adjacent tissue. These architectural distortions are difficult to detect thereby limiting the sensitivity of the screening procedure [18].

In response to these challenges, a range of software tools have been developed to help radiologists recognize subtle abnormalities in mammograms [7,19-23]. These tools typically use a common second reader model: the radiologist first examines the raw image and notes suspicious regions [24]. The tool then processes the image marking potentially suspicious regions and the results are compared.

Such systems have a significant drawback: they tend to have low specificity and so require nearly every image to be examined twice: once unaided, and then again to compare to the regions marked as suspicious by the software [25]. This is impractical for screening mammography where fewer than 1% of the images will have tumors. In that setting, the unintended consequence of CAD search routines is an increase the time required to report normal findings. In addition, increasing the number of prompts for review apparently does not guarantee an increase in accuracy [25].

Here we report the performance of a wavelet-map feature classifier (WFC), designed as a pre-sorting tool. The WFC identifies and removes normal images from the radiologists review queue, leaving those images with a higher probability of showing abnormalities. For this technique to be optimally safe, the algorithm is designed to perform at high sensitivity, detecting all or nearly all abnormalities; for it to be effective, it has sufficient specificity to remove enough normal images to usefully increase the relative frequency of suspicious images in the product queue.

The pre-screening algorithm was developed using the Digital Database for Screening Mammography (DDSM) database (http://marathon.csee.usf.edu/Mammography/Database.html) [26-28], a publicly available resource. A smaller unrelated Mammographic Images Analysis Society’s database (MIAS) database (http://peipa.essex.ac.uk/info/mias.html) [29] provided a confidence check that the algorithm was not over-specified. These data provided a useful proving ground for testing various incarnations of the algorithm.

The DDSM data set consisted of 1714 images, 1065 of which were classified as normal, in that they showed no abnormalities. The other 649 images showed some type of abnormality that would merit further study. These included: 119 benign calcifications, 120 cancerous calcifications, 213 benign masses and 197 cancerous masses. There was a range of tissue composition and breast size in the DDSM data set, making it representative of the variety of images that may be seen in a clinical setting.

The MIAS data set contained 303 images. There were 205 normal images and 98 images that showed some type of abnormality that included: 11 benign calcifications, 12 cancer-associated calcifications, 38 benign masses, 18 cancerous masses, and 19 architectural distortions (9 from benign masses and 10 from cancerous masses that were not directly visible). Again, the images were from a wide variety of patients, such that the tissues imaged varied widely in terms of breast size and tissue composition.

### The wavelet filter classifier process

The Wavelet Filter Classifier (WFC) proceeds in several discrete stages: regularizing the raw digital x-ray image, transforming it to produce scale maps, extracting features from the maps, classifying the features and generating the probability that the image contains some abnormality as an output.

### Regularizing the images

Digital mammograms were pre-processed [30] to reduce non-pathological variations between images, such as background noise, artifacts, and tissue orientation. All images were rescaled to 200 micron pixel resolution, and were padded or cropped to be 1024 × 1024 pixels, or 20.48 × 20.48 cm. The analysis presented here was restricted to medial-lateral views and the presentation of both breasts was adjusted to a single orientation.

The DDSM (and MIAS) mammograms were scanned from film images. As a result they contained label, noise and other artifacts that are not present in direct digital images. These artifacts were removed using a threshold and segmentation procedure. Otsu’s method [30] was used to determine the optimal pixel intensity threshold for distinguishing background and foreground (tissue) pixels. The segmented non-tissue regions were set to zero without changing pixel values within the tissue region. The processed images were rescaled to maximum pixel intensity.

### Wavelet decomposition

**Figure 1:**
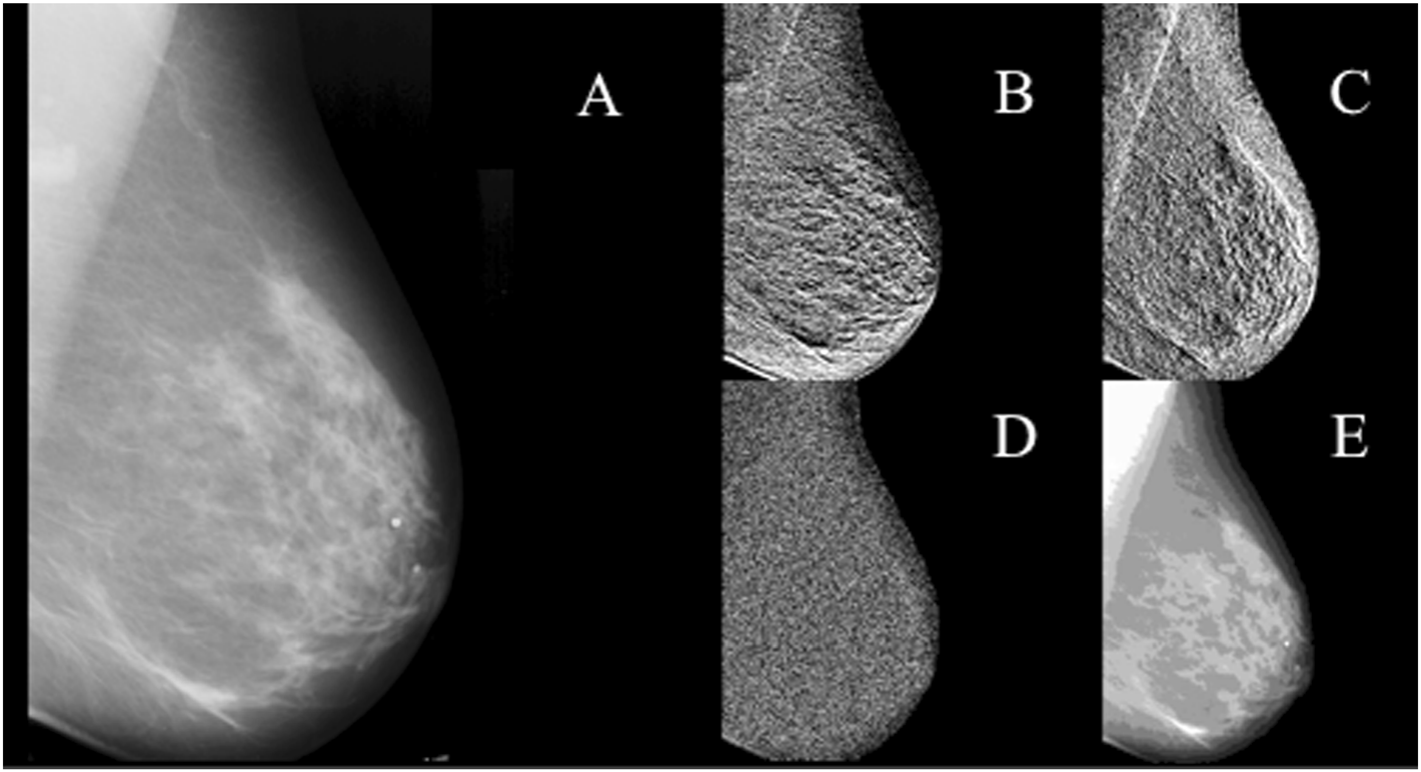
**Panel A.** Digitized film image masked to tissue. **Panels B - E** are Db2 wavelet coefficient maps at 2nd level of decomposition. **B**. horizontal detail, **C**. vertical detail, **D**. diagonal detail, **E**. approximation. Features were calculated from the tissue area.

The normalized mammograms were decomposed using 2D discrete wavelet transformation. This filtering process created four outputs: horizontal, vertical and diagonal detail maps from the high pass component and an approximation map using the low pass component applied vertically and horizontally. After each filter pass every second output pixel was kept. Figure 1 provides an example image and its feature maps generated at the second scale level using the Daubechies 2 wavelet. The high pass component produces scale maps at half the resolution of the parent image. They further emphasize features based upon how the image is sampled (horizontal, diagonal, vertical). The approximation image is the low-pass component and forms the input for the next scale transformation.

Eight decomposition levels were created in a serial process, applying the transformation to the approximation map to create four more maps. Since the approximation map had half the resolution of the input image, the wavelet sampled structures that were twice as large as in the original image. The set of all maps derived from a single original image formed a decomposition tree. The highest levels of the tree had the highest resolution and were most sensitive to structures with small spatial extent, while the lowest levels of the tree had the lowest resolution and were most sensitive to structures with large spatial extent.

Many wavelet bases are available, each with unique sampling characteristics. Several, including the Biorthogonal, Debuchies and Haar appeared promising for detecting subsets of the broad range of shapes and intensity gradients potentially associated with pathology [4,31-33]. Eleven wavelets were selected from these families, Haar, Db2, Db4, Db8, Bior1.5, Bior2.2, Bior2.8, Bior3.7 Bior4.4, Bior5.5, Bior6.8. The Haar wavelet is a square function that usefully interrogates sharp discontinuities. The other wavelets are more complex. The notation used suggests some of the features. For example, Db2 (Daubechies 2) is an orthogonal function that samples polynomials that have constant and linear scaling regions. The Bior1.5 describes a bi-orthogonal fifth order sampling function that requires a first order reconstruction algorithm.

The decompositions were initially performed in Matlab using the wavelet toolbox and later ported to C++ to improve computational efficiency. Moments of the mean generated from the output maps formed the input features for classification.

### Feature extraction

Four whole-image statistical features, mean, standard deviation, skewness and kurtosis of pixel intensity, were computed for each of the four wavelet-maps at each of the eight decomposition levels. This produced 132 scalar features for each of the eleven wavelet-bases applied to an x-ray image. The classification trials were restricted to using a combination of one, two or three features to avoid over-specifying the final classifier to the training set. Every combination of one, two or three features from the 132 member set were tested for every wavelet basis. The feature sets with the highest sensitivity for finding the images with known abnormalities were selected.

Mean and standard deviation are familiar metrics, skewness and kurtosis less so. The skewness value provides a measure of the asymmetry of a data distribution. Thus, the presence of a small number of unusually dark or bright pixels may alter skewness even when the mean and standard deviation values are not significantly affected. Here the skewness value may be sensitive to the representation of microcalcifications in an image. While these are only a few pixels in size they are unusually bright. Similarly, skewness may report the presence of bright (dense) masses. Since skewness measures the imbalance between the parts of the distribution above and below the mean, the presence of a dense mass will raise the skewness value relative to that found for a normal image.

Kurtosis reports the sharpness of the central peak of a distribution. Since it depends on the fourth power of the difference from the mean, it is highly sensitive to the addition of distant-valued points. Here, increasing numbers of bright microcalcification-containing pixels may be expected to raise kurtosis values. Interestingly, in some cases the kurtosis measure also detected masses. The post hoc rationale developed was based on the observation that when masses appear brighter than normal stromal tissue, they produced additional structure in wavelet maps at several scales. Adding intensity to normally dark pixels altered the kurtosis value sufficiently to distinguish it from the normal range. Of course for any feature, selection of wavelet bases and scale levels that correlate well with the shape of the anomaly was expected to provide the best differential. This was examined using eleven wavelet bases.

Selecting a subset of the candidate features added flexibility to the design of each individual classifier: for example, one classifier could use a feature subset sensitive to micro-calcifications while another could use a feature subset sensitive to masses.

Each classifier was limited to one wavelet basis and two of the four types of parameters generated from the maps. This reduced the feature pool size to 64. Combinations of these features were searched exhaustively to select the most effective combination.

The performance metric used to select the most effective feature subset was a weighted sum of the number of true positive classifications, NTP, and the number of true negative classifications, NTN. This score S was calculated as:

$$s=w\left(\mathrm{NTP}\right)+\left(1-w\right)\left(\mathrm{NTN}\right)$$

where *w* is a weighting factor that varies between zero and one. A high weighting factor favors a more sensitive classifier while a low weighting factor favors a more specific classifier. Since in this work, normal images were not subject to further analysis, the true positive fraction was maximized with a 0.995 weighting factor. When two feature subsets produced the same number of true positives the feature subset with the higher true negative fraction was selected.

The individual classifiers could also be designed to maximize detection of a specific abnormality (e.g. masses). To search for a single abnormality, NTP was replaced with the number of correctly classified images containing the specified abnormality, and NTN was replaced with the number correctly classified images of all other types. To ensure that other abnormalities were not missed by the complete system, the outputs of the individual classifiers were combined.

### Classification using feature subset and naïve Bayesian classifiers

The goal was to assist a reviewing physician make an informed decision in selecting images for further study. To do this, the classification scheme must provide a measure of the confidence that an image contains an abnormality. Since single naïve Bayesian classifiers do not generate confidence measures, a naïve Bayesian classifier network was constructed. The network’s performance classifying known images was used to calculate a classification confidence statistic. Training and testing was achieved using the leave-one-out cross-validation approach. Here, all but one of the samples were used to train the classifier, and the classifier is tested on the lone remaining sample. The overall performance of the classifier was measured by averaging the classification results when each sample in the data set was used as the test sample.

In all cases the selected scalar features calculated from an image’s wavelet maps formed the inputs. The network of classifiers was constructed by passing the normal and suspicious output images from one classifier into additional classifiers for further analysis; several network configurations were evaluated.

### Determining classification confidence from classifier network

The predicted confidence levels for a realistic distribution of normal and suspicious images were inferred from the results from a small data set after correcting for its inherent bias. In the DDSM data set [26-28], for example, 649 of the 1714 images were abnormal, this was a higher relative frequency than typically found in a screening clinic (1 in 20) [34]. To correct for this, the relative probability that a given input image was normal or suspicious, *P*_*i*_*(N)* and *P*_*i*_*(S)*, respectively was rescaled.

In the following discussion, lower case n and s refer to images classified as normal or suspicious, while upper case N and S refer to actual number of normal or suspicious images. If the number of normal images counted in a normal bin experimentally is *η*_*exp*_*(n,N)*, then the expected fraction of all images from a realistic distribution that are normal and are in the same bin, *F*_*real*_*(n,N)* was calculated as:

$$F_{\mathrm{real}}\left(n,N\right)=\eta_{\exp}\left(n,N\right)\frac{P_{\mathrm{real}}\left(N\right)}{T_{\exp}\left(N\right)}$$

where *P*_*real*_*(N)* was the probability of an image from the realistic distribution being normal and *T*_*exp*_*(N)* was the total number of normal images used in the experimental data set.

The realistic fraction of suspicious images in a normal bin, *F*_*real*_*(n,S)*, was similarly found from the experimentally counted number of suspicious images in the bin, *η*_*exp*_*(n,S)*.

The predicted confidence level for an image from a realistic distribution to be correctly placed into a certain normal bin, *C*_*real*_*(N)*, was calculated for each bin using the results measured from a small data set:

$$C_{\mathrm{real}}\left(N\right)=\frac{F_{\mathrm{real}}\left(n,N\right)}{F_{\mathrm{real}}\left(n,N\right)+F_{\mathrm{real}}\left(n,S\right)}=\frac{1}{1+\frac{1}{\alpha }\frac{\eta_{\exp}\left(n,S\right)}{\eta_{\exp}\left(n,N\right)}}$$

where *α* is a constant defined by:

$$\alpha =\frac{P_{\mathrm{real}}\left(N\right)T_{\exp}\left(S\right)}{P_{\mathrm{real}}\left(S\right)T_{\exp}\left(N\right)}$$

*α* characterizes the relative frequencies of normal and suspicious images in the experimental data set and in a realistic data set. For the DDSM data set [26-29] with 649 suspicious and 1065 normal images and for a clinic where 1 in 20 images are suspicious, *α* = 11.57. For the MIAS data set with 98 suspicious and 205 normal images and for a clinic where 1 in 20 images are suspicious, *α* = 9.08. A similar argument was used to calculate the confidence level (*C*_*real*_*(S)*) for an image from a realistic distribution to be correctly placed into a certain suspicious bin.

Confidence levels were calculated for the various classifier networks by counting the number of normal and suspicious images assigned to each output bin of the classifier network and using the value of *α* appropriate for the data set in question.

The relatively low number of suspicious images that occur in practice dominates the realistic confidence levels and makes all bins have a large confidence for containing normal images. To facilitate feature comparisons the theoretical case for an equal chance for an image to be normal or suspicious was also calculated. Thus, *C*_*real*_*(N)* gave the realistic likelihood that an image in a bin was normal, while *C*_*even*_*(N)* was useful for comparing the relative confidence levels of different bins when deciding which images are most likely normal. The mapping is monotonic, so bin ranking is the same using either a realistic or equal chance measure.

In summary, images were subjected to wavelet decomposition using a variety of bases and producing 32 scale maps per basis per image. Moments of the mean were calculated for each of the maps resulting in a total of 132 features per image per basis. A Bayesian classifier using leave-one-out cross validation was used to segregate the images into two groups: normal or suspicious. To enhance classification accuracy combinations of up to three features were evaluated. Where classifier networks were employed, a confidence level for the final classification was calculated.

## Results and discussion

### Feature selection

The moments of the mean features were evaluated on both the DDSM and MIAS databases to identify those features that best detected abnormal images. Single features provided sensitivity and specificity ranging from 89 to 94% and 17 to 38% respectively (Table 1). Better performance was observed using the DDSM than the MIAS database. The mean classification rate achieved on the DDSM database ranged from 50–60%. Whereas, the best performance on the smaller MIAS database did not exceed 47%. As a single feature, mean values provided the lowest sensitivity using either dataset. Standard deviation for DDSM and kurtosis for MIAS demonstrated the highest sensitivity. The best classification rate and sensitivity were obtained using a combination of features (Table 1). For example, using mean intensity and skewness together gave a sensitivity of 97%, and overall classification rate of 61%. Thus it appears that the sensitivity performance of the features, at least for some, was additive. These results were generally confirmed on the MIAS database, here the combination of mean and skewness achieved a sensitivity and classification rate of 92% and 47% respectively. In the case of this smaller database the individual components achieved sensitivities of 87% 92% respectively with classification rates near 44%.

**Table 1:** Mean performance of statistical features across all 11 wavelet bases tested

	**DDSM**			**MIAS**		
**Feature**^**§**^	Sensitivity* (%)	**Specificity**^**† **^**(%)**	**Classification rate**^**‡ **^**(%)**	**Sensitivity (%)**	**Specificity (%)**	**Classification rate (%)**
M	89.2	26.6	50.3	86.8	24.1	44.4
σ	94	27.6	52.8	87	27.7	46.9
S	90.8	29.4	52.7	91.9	20.6	43.6
K	92.8	23.7	49.8	93.5	16.8	41.6
M + σ	97.4	33.9	57.9	89.7	23.7	45
M + S	97.2	38.1	60.5	91.9	25.1	46.8
M + K	96.1	35.6	58.5	94	19.6	43.6
σ + S	95.6	29.1	54.3	93.2	23.1	45.8
σ + K	96.3	28.5	54.1	94.3	18	42.6
S + K	94.2	32.2	55.7	93.9	19.5	43.6

§M - mean, σ- standard deviation, S - skewness, K - kurtosis.

*Sensitivity is defined as TP/(TP + FN).

†Specificity is defined as TN/(TN + FP).

‡Classification rate is defined as (TP + TN)/(TP + TN + FP + FN).

The best single features obtained from the DDSM database were exhaustively tested for each wavelet over all scales and maps to identify the best combination of one, two or three features (Table 2). Sensitivities ranged from 93 to 99% with overall classification rates of 49 to 65%. The Bior 2.2 triplet combination of mean values from the horizontal detail map at scale 5 and the vertical detail map at scale 2, combined with the skewness value for the diagonal detail map at scale 2 achieved a sensitivity of 99% and an overall classification rate of 65%. In only one case, Bior 1.5, did a combination of just two features provide the best performance.

**Table 2:** Comparison of the performance of wavelet bases on the DDSM dataset

**Wavelet basis**^**§**^	**Best feature combination**			Sensitivity^* ^**(%)**	**Specificity**^**† **^**(%)**	**Classification rate**^**‡ **^**(%)**
Haar	M-h1	M-d1	S-h3	99.2	36.6	60.3
Db 2	M-h3	M-d8	S-h5	97.4	42.7	63.4
Db 4	M-h8	M-d1	S-h5	95.2	20.8	49
Db 8	M-h6	S-v8	S-d3	97.5	40.4	62
Bior 1.5	M-d4	S-h6	---	96.9	38.8	60.8
Bior 2.2	M-h5	M-v2	S-d2	98.8	44.8	65.2
Bior 2.8	M-d4	S-d2	S-a5	92.9	46.9	64.4
Bior 3.7	M-d4	S-h4	S-d4	98.9	28.1	54.9
Bior 4.4	M-h1	M-d4	S-d2	96.1	43	63.1
Bior 5.5	M-h6	M-d5	S-d2	98.5	38.1	61
Bior 6.8	M-v3	M-d4	S-d2	98	39	61.3

§Wavelet basis notation: Dbn where n describes the number of coefficients used in the wavelet. Db2 encodes polynomials with two coefficients, i.e. constant and linear components. Biorm.n where n describes the order for decomposition and m is the order used for reconstruction.

*Sensitivity is defined as TP/(TP + FN).

†Specificity is defined as TN/(TN + FP).

‡Classification rate is defined as (TP + TN)/(TP + TN + FP + FN).

The best triplet feature was selected for each wavelet.

A similar procedure was performed on the MIAS database (Table 3). Here the best features were found to be skewness and kurtosis. The Haar wavelet, using the triplet combination of skewness feature from the level 1 horizontal map combined with the kurtosis features from the approximation maps at levels 2 and 8, produced the best classification rate at 51%, but did so at the expense of sensitivity. All of the other wavelet bases tested provided higher sensitivity. The Bior2.8 basis using the doublet combination of the skewness value from the diagonal map at level 5 and the kurtosis value from the approximation map at level 4 provided the best combination of sensitivity (95%) and specificity (27%) and an overall classification rate of 49.2%. For this smaller dataset, four single and one double feature achieved the best performance for some wavelet bases. It may be noted in Table 3 that when single features exhibited good sensitivity they did so with a large specificity penalty.

**Table 3:** Comparison of the performance of wavelet bases on the MIAS dataset

**Wavelet basis**^**§**^	**Best feature combination**			Sensitivity^* ^**(%)**	**Specificity**^**† **^**(%)**	**Overall**^**‡ **^**classification rate (%)**
Haar	S-h1	K-a2	K-a8	90.8	32.7	51.5
Db2	K-a3			93.9	14.1	39.9
Db4	K-h5			94.9	9.3	37.0
Db8	S-h3	S-a4	K-d4	91.8	27.3	48.2
Bior 1.5	K-h3	K-a1	K-a8	94.9	13.7	39.9
Bior 2.2	K-a2			94.9	14.1	40.3
Bior 2.8	S-d5	K-a4		94.9	27.3	49.2
Bior 3.7	S-d6	K-d8	K-a7	93.9	23.9	46.5
Bior 4.4	K-h6	K-a2	K-a5	93.9	16.1	41.3
Bior 5.5	K-a1			93.9	14.1	39.9
Bior 6.8	S-h3	K-h7	K-a3	94.9	22.0	45.5

§Wavelet basis notation: Db*n* where *n* describes the number of coefficients used in the wavelet. Db2 encodes polynomials with two coefficients, i.e. constant and linear components. Bior*m.n* where *n* describes the order for decompositiona and *m* is the order used for reconstruction.

*Sensitivity is defined as TP/(TP + FN).

†Specificity is defined as TN/(TN + FP).

‡Classification rate is defined as (TP + TN)/(TP + TN + FP + FN).

The best triplet feature was selected for each wavelet.

The data had been normalized to 1024^2^ leaving open the possibility that the interpolation process may have influenced classifications rates. However, this was found not to be the case. A subset of the data was re-sampled to 256^2^ and to 512^2^ and classified using mean features from the Haar wavelet. The lower resolution images provided classification rates indistinguishable from the 1024^2^ resolution (not shown, see also [35]).

The results obtained (Tables 1, 2 and 3) suggested that no single combination of wavelet basis and feature would correctly classify all the images. Therefore, a network of classifiers was conceived in an attempt to achieve an acceptable classification rate.

**Figure 2:**
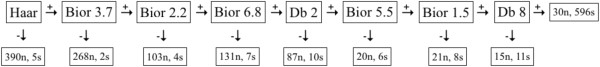
**A sequential classification network.** Images deemed suspicious were passed to a subsequent classifier for re-analysis. As images moved along the chain, the confidence that they were truly suspicious grew.

**Figure 3:**
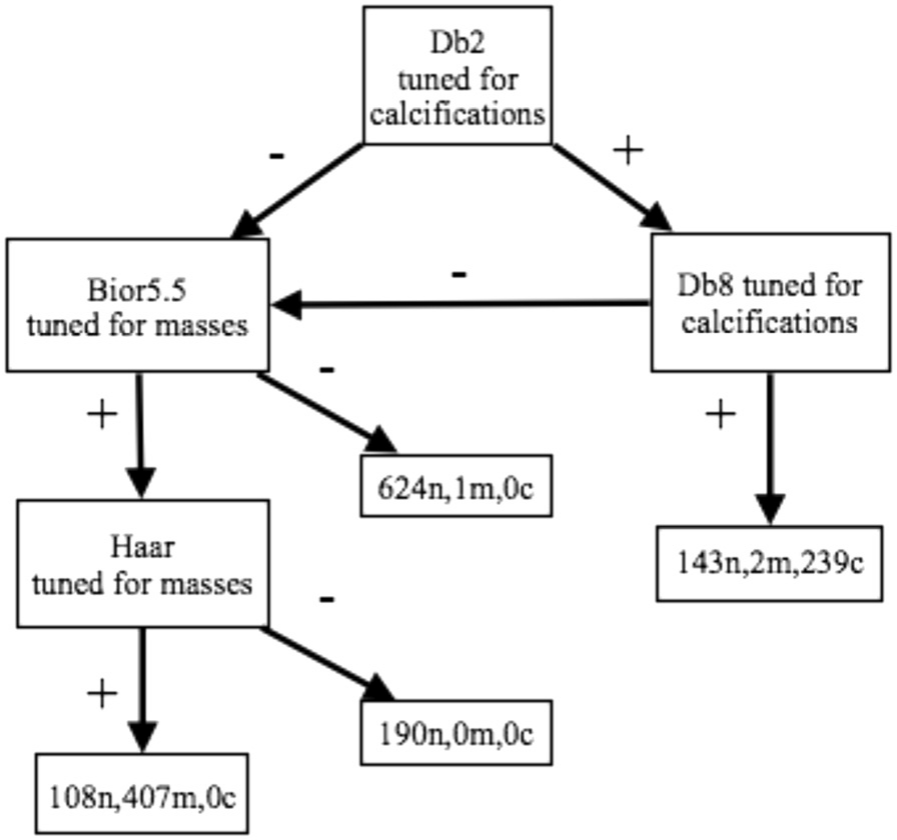
**A four-tap branched network.** Classifiers were tuned to preferentially detect calcifications or masses. Tuning refers to selecting the feature set to optimize sensitivity for the anomaly.

**Figure 4:**
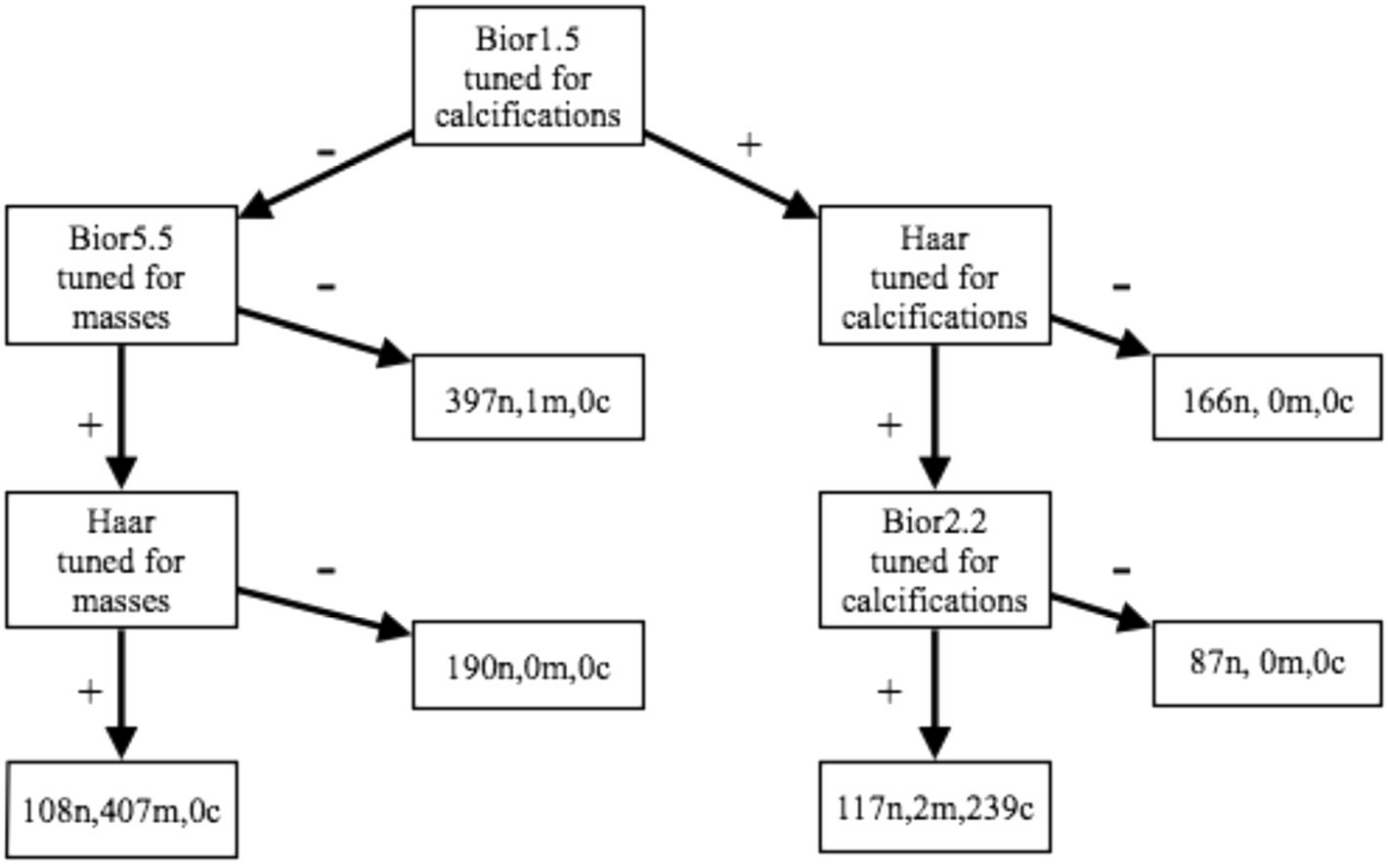
**A six-tap branched network.** Classifiers were tuned to preferentially detect calcifications or masses. Tuning refers to selecting the feature set to optimize sensitivity for the anomaly.

Two general network designs were developed and tested: 1) A sequential series of classifiers that passed images along a line of classifiers, removing them from the queue once found to be normal (Figure 2); 2) A branched network of classifiers initially tuned to just masses or just calcifications (Figures 3 and 4). The classifier features selected for the networks were those that had proven superior when tested alone. The findings from these approaches are presented below.

### Sequential network classifier

In the sequential configuration (Figure 2), an image’s wavelet map features (best set) were passed to the first classifier, images deemed normal were removed from the queue, while images classified as suspicious were passed on to the next classifier for re-analysis. Thus, the further an image passed along the chain before being found normal, the higher was its “suspicious” probability.

Classifiers selected for maximal sensitivity to any abnormality were organized in a sequential series. To increase the independence of the component classifiers, no wavelet basis was used more than once in a series. This criterion left eleven possible classifiers to choose among for the sequential design, one for each wavelet basis tested. An eight-member sequential series (Figure 2) was developed and the leave-one-out methodology was used for training and testing. Each individual classifier was the most sensitive for that wavelet basis. Tables 4 and 5 show the performance of the classifiers in the sequence that they were used on the DDSM and MIAS data respectively.

**Table 4:** Performance of sequential classifiers using the DDSM database

**Wavelet basis**^**§**^	**Classified normal**		**Classified suspicious**		**Confidence level (%)**	
	**Actually normal**	**Actually suspicious**	**Actually normal**	**Actually suspicious**	***C***_***even***_ ***(N)***	***C***_***even***_ ***(S)***
Haar	390	5	675	644	97.9	61
Bior 3.7	268	2	407	642	98.8	72.1
Bior 2.2	103	4	304	638	94	77.5
Bior 6.8	131	7	173	631	91.9	85.7
Db 2	87	10	86	621	84.1	92.2
Bior 5.5	20	6	67	615	67	93.8
Bior 1.5	21	8	45	607	61.5	95.7
Db 8	15	11	30	596	45.5	97

§Wavelet basis notation: Db*n* where *n* describes the number of coefficients used in the wavelet. Db2 encodes polynomials with two coefficients, i.e. constant and linear components. Bior*m.n* where *n* describes the order for decompositiona and *m* is the order used for reconstruction.

Suspicious images were passed to the next classifier in the chain.

**Table 5:** Performance of sequential classifiers on the MIAS database

**Wavelet basis**^**§**^	**Classified normal**		**Classified suspicious**		**Confidence level (%)**	
	**Actually normal**	**Actually suspicious**	**Actually normal**	**Actually suspicious**	***C***_***even***_ ***(N)***	***C***_***even***_ ***(S)***
Bior 2.8	56	5	149	93	84.3	56.6
Bior 6.8	23	0	126	93	100.0	60.7
Bior 3.7	9	1	117	92	81.1	62.2
Haar	50	5	67	87	82.7	73.1
Db 8	5	3	62	84	44.3	73.9

§Wavelet basis notation: Db*n* where *n* describes the number of coefficients used in the wavelet. Db2 encodes polynomials with two coefficients, i.e. constant and linear components. Bior*m.n* where *n* describes the order for decompositiona and *m* is the order used for reconstruction.

Suspicious images were passed to the next classifier in the chain.

For the DDSM data, the Haar based classifier correctly identified 390 of the 1065 normal images in the set and misidentified 5 of the 649 suspicious images as normal. This provided a confidence level, using an equal prior probability of normal or suspicious, of 97.9% for normal and 61% for suspicious. Images classified as suspicious were passed down the chain configured with Biorthogonal and Daubechies based classifiers. After stage five in the chain, the confidence that an image classified as normal, was normal, declined sharply. This implied that the incidence of type II error (false negative) rose at this stage and beyond. Considering the emphasis placed on detection in this study, the data suggested that this eight member sequential network might be terminated at stage 5 to maintain high sensitivity. Overall, the DDSM-trained sequential network achieved 91.8% sensitivity for abnormal images with a specificity of 97.2%. Eight percent of the positive images escaped detection.

Re-evaluation on the MIAS-trained network (using different features) achieved 88.8% sensitivity to abnormal images with a specificity of 67.3% at stage 5. These results were very encouraging and led to the second network approach.

### Network tuned for particular abnormalities

The alternative embodiment used classifiers that were tuned to detect specific types of abnormalities, either masses or calcifications. The goal was to determine if performance might be improved by deploying specialized classifiers. Images were first passed through several classifiers looking for one type of abnormality; if they were not suspicious for it, they were passed on to several classifiers looking for the other type of abnormality. These classifiers, with more specific targets, had potentially higher sensitivities. Figures 3 and 4. show two networks designed in this way. The number of images that are normal (n), show calcifications (c), or show masses (m) are listed at each stage of the network with the appropriate letter label. The wavelet features selected were those that had best identified the anomaly as a single feature classifier.

The tuned classifier networks were configured with four or six output taps. This offered the additional potential to distinguish among normal, calcifications and masses. The network selected calcifications first. The four-tap network (Figure 3) used the Db2 wavelet feature tuned for calcifications. Suspicious images were passed to a Db8 classifier also tuned for calcifications. Normal images from the Db8 classifier went to the queue with normal images from the Db2 classifier, to be reexamined using Bior5.5 and Haar classifiers tuned for masses. The Db8 classifier output on the calcifications leg, was a bin with all the images containing calcifications, a few masses and some normal images. On the masses leg of this network the suspicious tap contained most of the masses (all but one), no calcifications and some normal images. The normal output bins on this leg contained 814 of the 1065 normal images, one mass and no calcifications. This configuration achieved 99.8% sensitivity, a specificity of 76.4% and a classification rate of 86.1%.

For the six-tap network, classification began with the Bior1.5 (Figure 4). Suspicious images from this were passed successively to Haar and Bior2.2 classifiers both tuned for calcifications. The suspicious output on this leg was a bin containing all the calcifications, 2 masses and 117 normal images. There were two normal output bins on this leg, these bins contained only normal images. The normal output from the Bior1.5 was passed to Bior5.5 and Haar classifiers tuned for masses. On this leg the classifiers identified all but one of the masses. The two normal bins contained no calcifications and a single mass. This configuration provided a sensitivity of 99.8%, a specificity of 78.9% and an overall classification rate of 86.8%. This configuration successfully removed 840 of the 1065 images from the suspicious bin. To achieve this result, the penalty was one incorrectly classified mass-containing image.

When similar networks were evaluated on the MIAS data set equivalent results were obtained (not shown). Here again, in the four-tap configuration calcifications were identified first, then masses. The six-tap configuration searched for masses first, then calcifications. Using this smaller dataset the four-tap configuration achieved 100% sensitivity and 46.3% specificity with an overall classification rate of 63.7%. The six-tap network also achieved 100% sensitivity, and 65% specificity. Here the overall classification rate achieved was 76.6%.

Table 6 collects the results for each of the network configurations and provides the realistic confidence for the classification. The sensitivity of the systems was such that even in a realistic data set, when the incidence of abnormality was 5%, it is likely that all abnormalities will be detected.

**Table 6:** Performance of branched network classification

**Dataset**	**TP**	**TN**	**FP**	**FN**	**Sensitivity**	**Specificity**	**Class. rate**	***C***_***real***_ ***(N)***	***C***_***real***_ ***(S)***
DDSM									
four-tap	748	814	251	1	1.0	0.764	0.861	0.975	1
six-tap	648	840	225	1	1.0	0.789	0.868	0.979	1
MIAS									
4 tap	98	95	110	0	1.0	0.463	0.637	0.975	1
6 tap	98	134	71	0	1.0	0.654	0.766	0.979	1

The networks also achieved a strong segmentation between images with calcifications and images with masses. The data is collected in Table 7 where it is evident that the networks were perfectly sensitive for calcifications although perhaps less efficient with the smaller MIAS data set. It is tempting to speculate that this is due to under-representation of the range of normal mammogram types in MIAS.

**Table 7:** Segmentation of mammograms containing masses from those containing calcifications

**Dataset**	**T-Norm**	**F-Norm**	**T-Mass**	**F-Mass**	**T-Calc**	**F-Calc**	***C***_***even ***_ ***(Norm)***	***C***_***even ***_ ***(Mass)***	***C***_***even ***_ ***(Calc)***
DDSM									
four- tap	814	1	407	108	239	145	0.764	0.993	1.000
six- tap	840	1	407	1078	239	119	0.770	0.993	1.000
									
MIAS									
4 tap	95	0	71	86	23	28	0.463	0.947	1.000
6 tap	134	0	75	66	23	5	0.654	1.000	1.000

The networks were designed conservatively; each wavelet classifier was configured for maximum sensitivity. A more aggressive design could have removed more normal images, but may have sacrificed overall sensitivity; that was not considered an acceptable tradeoff.

The classifiers developed in this paper offer a useful approach for binary classification of mammographic x-ray images. In practice, an analyst could use the WFC, tuned to a confidence threshold of their choosing, to remove or re-prioritize normal images. This pre-screening technique should improve subsequent detection of those few images showing abnormalities that merit further analysis [5,36].

For an algorithm to be effective and optimally safe as a preliminary screening tool, it must be able to correctly identify a significant number of normal images while minimizing the number of suspicious images that are incorrectly identified as normal. That is, the algorithm must offer sensitivity higher than current clinical levels, which have been estimated to be between 75% and 90% [2,3,5,34,36-38], while offering a non-negligible specificity. Both branched networks tested in this study achieved sensitivity superior to current clinical performance.

## Conclusion

An x-ray mammogram image analysis system [39] was tested on two independent data sets to measure its ability to identify suspicious images that may merit further study by a human expert. The system operated in several steps: first, an image was pre-processed to reduce background noise and artifacts; second, the image was decomposed into a set of maps at different scale levels using a 2D discrete wavelet transform; third, whole-image statistical features were measured from each map and the best triplet of these features was input into naïve Bayes classifiers to determine if an image is normal or suspicious; fourth, several classifiers were chained together to calculate confidence levels from the normally hard classifiers.

Three network designs were tested here: a sequential series of classifiers, a vote-taking scheme of classifiers, and networks where individual classifiers were tuned to detect only calcifications or only masses. All of the networks were designed with sensitivity as the top priority over specificity, since the system is designed to be a first pass for images, so any abnormal images missed by the algorithm would not be likely re-examined by a human expert. All the networks tested provided higher sensitivity than is typically achieved in the screening clinic. Removing a large fraction of normal images from the review queue will reduce the volume of cases that must be examined and, at least statistically, should improve detection of pathology. In the best-case scenario reported here, pre-sorting the images doubled the prior probability of disease in the sorted database.

Once sensitivity is maximized, the effectiveness of a system is governed by its specificity. Here the expert reader excels, typically achieving greater than 95% specificity. The combination of a highly sensitive pre-screening tool and an expert breast screener promises to significantly enhance the overall performance of the typical screening program.

## Competing interests

Financial competing interests:

EJK and MGB hold United States Patent US 2010/0310183 that deals, in part, with the material presented in this manuscript. See reference [39].

Non-financial competing interests:

The authors declare that there are no non-financial competing interests.

## Authors’ contributions

EJK study concept, data analysis/interpretation, prepared manuscript. MGB study concept, data analysis/interpretation, reviewed manuscript, Matlab and C++ programming. KCP study concept, data analysis/ interpretation, reviewed manuscript, feature generation and matrix size experiments. All authors are responsible for the content of the paper and approved the final draft.

## Pre-publication history

The pre-publication history for this paper can be accessed here:

http://www.biomedcentral.com/1471-2342/13/43/prepub
